# Prompt and Intensive Antiviral Chemoprophylaxis in Nursing Home Influenza Outbreaks

**DOI:** 10.1001/jamainternmed.2026.0401

**Published:** 2026-03-30

**Authors:** Joe B. B. Silva, Han-Chih T. Hsieh, Chanelle J. Howe, Stefan Gravenstein, Laura A. Reich, Andrew R. Zullo

**Affiliations:** 1 Department of Health Services, Policy, and Practice, Brown University School of Public Health, Providence, Rhode Island; 2 Department of Epidemiology, Brown University School of Public Health, Providence, Rhode Island; 3 Center for Epidemiologic Research, Brown University School of Public Health, Providence, Rhode Island; 4 Department of Epidemiology, Boston University School of Public Health, Boston, Massachusetts; 5 Division of Geriatrics and Palliative Medicine, Warren Alpert Medical School of Brown University, Providence, Rhode Island; 6 Providence Veterans Affairs Medical Center, Providence, Rhode Island; 7 Center for Gerontology and Healthcare Research, Brown University School of Public Health, Providence, Rhode Island

## Abstract

**Importance:**

Influenza outbreaks in nursing homes (NHs) can cause high morbidity and mortality. Antiviral chemoprophylaxis with oseltamivir is recommended, yet optimal implementation strategies remain unclear.

**Objective:**

To examine whether initiating antiviral chemoprophylaxis for 70% or more of eligible NH residents within 2 days of influenza outbreak detection is associated with lower all-cause mortality and hospitalization at 14 and 30 days.

**Design, Setting, and Participants:**

Retrospective cohort study using a sequential cluster-randomized target trial emulation and randomize-censor-weight approach for influenza outbreaks (September 1, 2018–May 31, 2022) in 12 US NH corporations. Eligibility criteria were age 18 years or older, present on the outbreak-detection day, no antiviral use in the preceding 7 days, no influenza in the past 14 days, and complete baseline data. Residents were followed up until hospitalization or death, an NH discharge to a nonacute-care location, or the end of follow-up. Data were analyzed from February 2023 to January 2026.

**Exposures:**

Intensive antiviral chemoprophylaxis with oseltamivir (≥70% of eligible residents within 2 days of outbreak detection) or nonintensive antiviral chemoprophylaxis (0% to <70% of eligible residents).

**Main Outcomes and Measures:**

Outcomes were all-cause death and hospitalizations within 14 and 30 days of outbreak detection. Discrete-time hazard models with pooled logistic regression were applied to estimate weighted risks, risk differences (RDs), and risk ratios (RRs).

**Results:**

Among 404 outbreaks in 318 NHs, 35 086 resident-trial observations (29 683 residents; median age 78 [IQR, 68- 86] years; 60% women; 81% White; 76% vaccinated) met eligibility criteria. Intensive oseltamivir prophylaxis was randomized to 17 155 observations; 17 931 were randomized to nonintensive care. At 14 days, intensive prophylaxis vs nonintensive yielded an RD of –0.06% (95% CI, −0.73% to 0.93%) and an RR of 0.96 (95% CI, 0.56-1.57) for death, and an RD of –0.96% (95% CI, −1.78% to −0.19%) and an RR of 0.79 (95% CI, 0.64-0.96) for hospitalization. At 30 days, the hospitalization differences persisted but were less precise and there continued to be no difference in death.

**Conclusions and Relevance:**

Study results suggest that clinicians should initiate antiviral chemoprophylaxis for at least 70% of eligible NH residents within 2 days of outbreak detection to lower risk of hospitalization.

## Key Points

**Question:**

Is initiation of antiviral chemoprophylaxis with oseltamivir for 70% or more of eligible nursing home (NH) residents within 2 days of outbreak detection associated with lower 14-day and 30-day mortality and hospitalization compared with a nonintensive approach?

**Findings:**

In this cohort study of 404 influenza outbreaks across 318 NHs with 35 086 resident-trial observations using a sequential target trial emulation and the randomize-censor-weight approach, hospitalization but not death was lower at 14 days post outbreak in NHs that implemented intensive antiviral chemoprophylaxis; 30-day estimates were directionally similar but less precise.

**Meaning:**

Results of this study suggest that clinicians should promptly initiate antiviral chemoprophylaxis in at least 70% of NH residents within 2 days of an influenza outbreak to markedly reduce influenza-related hospitalizations.

## Teaser

This cohort study assesses the association between different nursing home antiviral chemoprophylaxis strategies and the risks of all-cause death and hospitalization.

## Introduction

Influenza outbreaks in nursing homes (NHs) pose a substantial threat to older adults, often resulting in morbidity and mortality. The Centers for Disease Control and Prevention (CDC) and the Infectious Diseases Society of America (IDSA) recommend prompt postexposure prophylaxis, also termed chemoprophylaxis or prophylaxis with oseltamivir, for all residents who are not ill to limit influenza spread in NHs.^1,2^

Early trials left the net benefits of oseltamivir uncertain,^3,4,5,6,7,8^ yet CDC/IDSA guidelines still give chemoprophylaxis a strong (A-III) recommendation based on limited low-quality data.^2^ Residents live in close quarters, share staff, and often cannot be effectively isolated.^9^ Therefore, near-universal and rapid chemoprophylaxis initiation may improve outbreak control. However, to our knowledge, no study has defined the coverage (eg, ≥70% of residents) or speed to curb mortality and hospitalization. Prior observational studies included small outbreaks with widely varying coverage (20%-100%) and initiation delays of 2 to 10 days.^10,11,12,13,14,15^ High-quality evidence on optimal coverage thresholds and timing is therefore needed to guide practice.

We conducted an observational study to examine the association between different NH antiviral chemoprophylaxis strategies and the risks of all-cause death and hospitalization. Because large well-powered cluster-randomized trials of chemoprophylaxis are lacking and clinical implementation unfolds over days, we used target trial emulation to specify a hypothetical cluster-randomized pragmatic trial and emulate it in longitudinal NH electronic health record (EHR) data, estimating the 14-day and 30-day risks of death and hospitalization under alternative chemoprophylaxis strategies.

## Methods

### Study Design and Data Sources

This retrospective cohort study used proprietary EHR data from 12 US NH corporations during the study period, September 1, 2018, through May 31, 2022. Data were analyzed from February 2023 to January 2026. The study period aligns with influenza seasons defined by the availability of NH EHR data.^16^ The target trial compared administration of chemoprophylaxis with oseltamivir to 70% or more of eligible residents within 2 days of outbreak detection vs providing no chemoprophylaxis or chemoprophylaxis for more than 0% but less than 70% of residents. eTable 1 in Supplement 1 provides specification of the target trial emulation process. The EHR database contained resident demographics, diagnosis codes, medication orders, electronic medication administration records (eMAR), and minimum data set (MDS) clinical assessments. Information on resident race and ethnicity was obtained from self-reported race and ethnicity data collected during MDS assessments and, if missing, from administrative records in the NH EHR. No modifications to the race and ethnicity data were made by the investigators. Race and ethnicity data were reported to describe the study population. The study was approved by the Brown University Institutional Review Board, which waived informed consent because the research involved minimal risk to participants, the impracticality of conducting without a waiver due to large sample size, and use of secondary data. This study followed the Strengthening the Reporting of Observational Studies in Epidemiology (STROBE) reporting guideline.

### Population

The target trial included NHs that had a detected influenza outbreak. We defined outbreaks by the detection of a second incident influenza case within 72 hours of the first, and NHs became eligible for enrollment on that day (ie, baseline). Incident influenza cases were defined as any identified following a resident’s admission. NHs included in the target trial must have had complete covariate information, 30 or more residents present in the facility at baseline, and baseline covariate data for 50% or more of residents present at baseline. We required 30 or more residents to ensure reliable estimates of chemoprophylaxis utilization. We assumed that missing data for NHs and residents occurred at random.^17^ NH trial enrollment began September 1, 2018. Residents eligible to contribute data on outcomes during follow-up met the following criteria: present in the NH at baseline, aged 18 years or older, and had complete MDS covariate information. For chemoprophylaxis eligibility, residents could not have received any antiviral medication in the past 7 days or influenza identified in the past 14 days. eFigure 1 in Supplement 1 provides a detailed inclusion and exclusion of NHs and residents.

The target trial emulation applied similar eligibility criteria, except NHs were required to have no influenza outbreak 14 days prior to baseline and have an active eMAR at baseline to ascertain chemoprophylaxis utilization. To define outbreaks as distinct events, we required a 2-week gap after the end of an outbreak before new incident cases within a 72-hour window could establish another outbreak. We considered residents with a recorded age of 110 or more years at baseline ineligible due to concerns about the accuracy of the recorded age.

### Intervention and Comparator

In the target trial, we would cluster-randomize eligible NHs (ie, all residents in the same NH received the same treatment) at baseline in an open-label manner to 1 of the 2 treatment strategies without blinding: intensive antiviral chemoprophylaxis, initiating chemoprophylaxis with oseltamivir at any dose for 70% or more of residents eligible to receive chemoprophylaxis within the first 2 days of baseline or nonintensive antiviral chemoprophylaxis, not initiating chemoprophylaxis or initiating chemoprophylaxis for greater than 0% but less than 70% of residents and continuing that approach for the duration of follow-up. We chose the 70% threshold because of the impracticability of near-universal implementation in practice (eg, due to contraindications, refusals, comfort-focused care, or residents temporarily offsite) and simulation studies suggesting that resident coverage at or above the feasible-to-achieve 70% level produces a meaningful reduction in influenza transmission and hospitalizations in NH outbreaks.^18^ Other infection control measures and resident therapies remained at staff or clinician discretion. Chemoprophylaxis was measured daily as the percentage of eligible residents receiving oseltamivir. In the emulation, the treatment strategies were the same, but we ascertained oseltamivir utilization from eMAR records.

### Study Outcomes and Follow-Up

In the target trial, we examined all-cause death and all-cause hospitalization as the primary outcomes. We followed up individuals from baseline (randomization of the NH on the day of outbreak detection) until the first occurrence of an outcome event (each evaluated separately) discharge from the NH to a location other than an acute care hospital, or end of follow-up: 14 days or 30 days after baseline. We chose 14-day and 30-day follow-ups because one reflects the recommended duration of chemoprophylaxis and the other provides a practical window to assess resident outcomes.^1,19,20^ Follow-up ended May 31, 2022.

The emulation used the same outcomes and follow-up as the target trial, but deaths occurring outside the NH were not consistently measured.

### Statistical Analysis

Consistent with the randomize-censor-weight method, to emulate the treatment assignment of the target trial in our observational study, we randomized NHs in a given outbreak analytically to either of the 2 treatment strategies at baseline. In the target trial, this randomization would have been part of the data-generating process, but in our observational study, this random assignment is an analytic device since NHs actually had the ability to respond to outbreaks at their discretion. Residents were censored if the NH in which they resided deviated from the assigned treatment strategy, with a 2-day postoutbreak detection grace period. The causal estimands were the observational analogues of the per-protocol effect. We focused on the per-protocol effect because our scientific question concerned the outcomes associated with successful implementation of a rapid high-coverage chemoprophylaxis response vs a nonintensive response.

To increase sample size, eligibility criteria were applied on each day of each influenza season to identify eligible NHs on the day of outbreak detection as well as residents present in the facility on that day, thereby creating a sequence. NHs and residents could participate in as many of the sequential trials as they met eligibility for. The primary unit of analysis was therefore the resident-trial (ie, a unique instance of a resident present during a particular influenza outbreak in an NH).

Inverse probability of censoring weights (IPCW) were estimated to account for censoring due to deviation from the assigned treatment (eFigure 2 in Supplement 1) using the following covariates: tertile of Alzheimer disease and related dementias (ADRD) prevalence, tertile of the count of prior influenza tests ordered during the week prior to baseline, whether the outbreak was first to occur in the NH during a given season, and a time-varying indicator of whether additional cases of influenza, beyond the initial 2, had been identified. Pooled logistic regression models were used to estimate the nonstabilized IPCW for the intensive and nonintensive arms separately. The distributions of the IPCW are available in eTable 2 in Supplement 1. The model for the nonintensive group also included time and product terms between day of follow-up and all other covariates. Finally, the IPCW were incorporated into discrete-time hazard models fit using pooled logistic regression. These models included outcomes regressed on treatment strategy, linear and quadratic polynomials for time, and product terms between time and treatment strategy. The cumulative product of the model estimated probabilities was used to estimate 14-day and 30-day IPCW-weighted risks, risk differences (RDs), and risk ratios (RRs).^21^ Because of within-NH clustering, we used a nonparametric Poisson-weighted bootstrap at the NH level with 500 replicates to generate valid 95% CIs.^22,23^

We conducted 3 sensitivity analyses. First, we redefined the intensive antiviral chemoprophylaxis treatment strategy as implementing 70% or more chemoprophylaxis within 7, rather than 2, days of outbreak detection. Second, we examined alternative treatment strategies (≥50%, ≥60%, ≥80%, and ≥90% NH-level chemoprophylaxis coverage within 2 days). Third, we repeated our primary analysis among only those residents aged 65 years or older.

Data preparation was conducted using SAS version 9.4 TS1M9 (SAS Institute). All statistical analyses were conducted using R version 4.3.1 (R Foundation for Statistical Computing).

## Results

### Study Population

We identified 404 influenza outbreaks across 318 NHs, generating 35 086 resident-trials (29 683 unique residents). Of these, 17 155 were in the intensive chemoprophylaxis group and 17 931 were in the nonintensive group. The median number of residents per NH at the start of follow-up was 83 (IQR, 54-112). Across all residents, the median age was 78 (IQR, 68-86) years, 60.1% were female, 81.2% were non-Hispanic White, and 76.1% were vaccinated for influenza (Table 1).

**Table 1.** Baseline Characteristics of Eligible Residents in Nursing Homes With Detected Influenza Outbreaks, 2018-2019 to 2021-2022 Influenza Season^a^

Characteristic^b^	All (N = 35 086)	Nonintensive response (n = 17 931)	Intensive response (n = 17 155)	Absolute standardized mean difference
**Resident characteristic**				
Age, median (IQR), y	78 (68-86)	78 (68-87)	77 (67-86)	0.045
Sex				
Male	13995 (39.9)	7042 (39.3)	6953 (40.5)	0.026
Female	21 091 (60.1)	10 889 (60.7)	10 202 (59.5)	
Race and ethnicity^c^				
Asian or Other Pacific Islander	1154 (3.3)	668 (3.7)	486 (2.8)	0.061
Hispanic	1310 (3.7)	658 (3.7)	652 (3.8)	
Native American or Alaska Native	187 (0.5)	93 (0.5)	94 (0.5)	
Non-Hispanic Black	3933 (11.2)	1909 (10.6)	2024 (11.8)	
Non-Hispanic White	28 502 (81.2)	14 603 (81.4)	13 899 (81.0)	
ADL scale, median (IQR)^d^	18 (13-20)	18 (13-20)	18 (13-20)	0.009
Mortality Risk Score, median (IQR)^e^	4 (2-6)	4 (3-6)	4 (2-6)	0.089
Influenza vaccination	26 691 (76.1)	13 839 (77.2)	12 852 (74.9)	0.053
**Nursing home characteristic**				
First outbreak identified in nursing home during influenza season	31 344 (89.3)	16 258 (90.7)	15 086 (87.9)	0.088
Additional cases of influenza detected	14 214 (40.5)	6847 (38.2)	7367 (42.9)	0.097
Orders for influenza testing in prior week				
0-1	12 787 (36.4)	6775 (37.8)	6012 (35.0)	0.149
2-3	9424 (26.9)	4240 (23.6)	5184 (30.2)	
4-64	12 875 (36.7)	6916 (38.6)	5959 (34.7)	
Residents with ADRD, %				
0-28.6	10 863 (31.0)	5336 (29.8)	5527 (32.2)	0.191
28.8-43.1	12 450 (35.5)	5810 (32.4)	6640 (38.7)	
43.1-98.4	11 773 (33.6)	6785 (37.8)	4988 (29.1)	

Abbreviations: ADL, activities of daily living; ADRD, Alzheimer disease and related dementias; MDS, minimum data set.

^a^ All values are for resident-trial observations at baseline (outbreak detection). Residents could contribute multiple observations across distinct outbreaks. Therefore, the unit of analysis is the resident-trial (a resident’s observation within a given outbreak trial).

^b^ Characteristics are reported as number (%) unless otherwise noted.

^c^ Information on resident race and ethnicity was obtained from self-reported race and ethnicity data collected during MDS assessments and, if missing, from administrative records in the nursing home electronic health record. No modifications to the race and ethnicity data were made by the investigators. Race and ethnicity data were reported to describe the study population.

^d^ Physical function was measured using the Morris 28-point ADL scale, categorized from independent to limited required assistance (0-14), extensive assistance required (15-19), and extensive dependency (20-28).

^e^ Mortality risk was measured using the MDS 3.0 Mortality Risk Score, scored 1-39 based on measures found in MDS assessments related to cognitive and physical function, diagnoses, and health conditions.

### All-Cause Mortality

Among 17 931 nonintensive chemoprophylaxis resident-trials, the weighted risk of death at 14 days was 1.65% (95% CI, 1.43%-1.97%) and at 30 days was 3.02% (95% CI, 2.64%-3.56%) (Table 2; eTable 3 in Supplement 1). Of the 17 155 intensive chemoprophylaxis resident-trials, the weighted risk of death was 1.59% (95% CI, 0.97%-2.52%) and at 30 days was 3.20% (95% CI, 1.97%-5.25%). Comparing intensive chemoprophylaxis to nonintensive, the 14-day RD was −0.06% (95% CI, −0.73% to 0.93%) and the RR was 0.96 (95% CI, 0.56-1.57). The 30-day RD was 0.18% (95% CI, −1.25% to 2.30%) and the RR was 1.06 (95% CI, 0.62-1.79). Figure, A shows the 30-day weighted survival curves by treatment strategy.

**Table 2.** Per-Protocol Analysis of 14-Day and 30-Day Risks of Death and Hospitalization Comparing Intensive (≥70% Within 2 Days) vs Nonintensive Antiviral Chemoprophylaxis Responses

Outcome and follow-up	% (95% CI)			Risk ratio (95% CI)
	Nonintensive risk	Intensive risk	Risk difference	
Death				
14 d	1.65 (1.43 to 1.97)	1.59 (0.97 to 2.52)	−0.06 (−0.73 to 0.93)	0.96 (0.56 to 1.57)
30 d	3.02 (2.64 to 3.56)	3.20 (1.97 to 5.25)	0.18 (−1.25 to 2.30)	1.06 (0.62 to 1.79)
Hospitalization				
14 d	4.54 (4.06 to 5.11)	3.58 (3.01 to 4.14)	−0.96 (−1.78 to −0.19)	0.79 (0.64 to 0.96)
30 d	7.33 (6.63 to 8.13)	6.26 (4.81 to 7.46)	−1.07 (−2.70 to 0.38)	0.85 (0.64 to 1.05)

**Figure.**
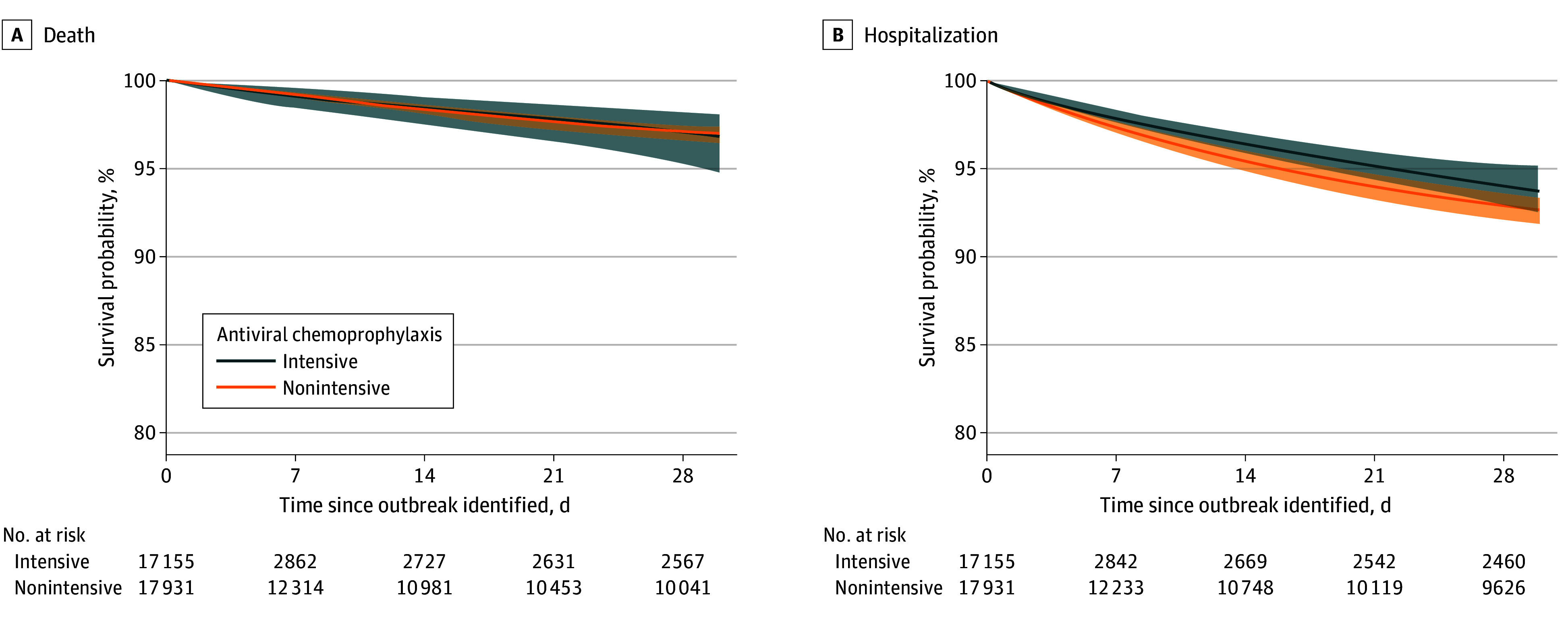
Weighted 30-Day Survival Curves Comparing Intensive vs Nonintensive Antiviral Chemoprophylaxis Responses Intensive antiviral chemoprophylaxis was administering chemoprophylaxis with oseltamivir to 70% or more of eligible residents within 2 days of influenza outbreak detection. Nonintensive antiviral chemoprophylaxis was providing no chemoprophylaxis or chemoprophylaxis for more than 0% but less than 70% of eligible residents. Shaded regions denote 95% CIs derived from a nonparametric Poisson-weighted nursing home-level bootstrap with 500 replicates.

### All-Cause Hospitalization

Among the nonintensive chemoprophylaxis resident-trials, the weighted risk of hospitalization at 14 days was 4.54% (95% CI, 4.06%-5.11%) and at 30 days was 7.33% (95% CI, 6.63%-8.13%) (Table 2; eTable 3 in Supplement 1). Among intensive chemoprophylaxis resident-trials, the weighted risk of hospitalization at 14 days was 3.58% (95% CI, 3.01%-4.14%) and at 30 days was 6.26% (95% CI, 4.81%-7.46%). Comparing intensive chemoprophylaxis to nonintensive, at 14 days, the RD was −0.96% (95% CI, −1.78% to −0.19%) and the RR was 0.79 (95% CI, 0.64-0.96). At 30 days the RD was −1.07% (95% CI, −2.70% to 0.38%) and the RR was 0.85 (95% CI, 0.64-1.05). Figure, B shows the 30-day weighted survival curves by treatment strategy.

### Sensitivity Analysis

Results from modification of the treatment strategy to intensive chemoprophylaxis within 7 days are presented in Table 3. Under this alternative strategy, estimates were generally closer to the null and imprecise. At 14 days, the RR and RD were 0.81 (95% CI, 0.32 to 1.11) and −0.32% (95% CI, −1.16 to 0.17) for death and 1.06 (95% CI, 0.89 to 1.45) and 0.29% (95% CI, −0.53 to 1.93) for hospitalization. At 30 days, the RR and RD were 1.17 (95% CI, 0.81 to 1.35) and 0.50% (95% CI, −0.66 to 0.96) for death and 0.97 (95% CI, 0.79 to 1.43) and −0.24% (95% CI, −1.58 to 3.01) for hospitalization.

**Table 3.** Per-Protocol Sensitivity Analysis of 14-Day and 30-Day Risks of Death and Hospitalization Comparing Intensive (≥70% Within 7 Days) vs Nonintensive Antiviral Chemoprophylaxis Responses

Outcome and follow-up	% (95% CI)			Risk ratio (95% CI)
	Nonintensive risk	Intensive risk	Risk difference	
Death				
14 d	1.65 (1.43 to 1.96)	1.33 (0.52 to 1.71)	−0.32 (−1.16 to 0.17)	0.81 (0.32 to 1.11)
30 d	3.02 (2.62 to 3.50)	3.52 (2.59 to 3.89)	0.50 (−0.66 to 0.96)	1.17 (0.81 to 1.35)
Hospitalization				
14 d	4.55 (4.09 to 5.13)	4.84 (4.13 to 6.45)	0.29 (−0.53 to 1.93)	1.06 (0.89 to 1.45)
30 d	7.34 (6.63 to 8.17)	7.10 (5.90 to 10.20)	−0.24 (−1.58 to 3.01)	0.97 (0.79 to 1.43)

In analyses using alternative thresholds (Table 4) to define an intensive chemoprophylaxis response (≥50%, ≥60%, ≥80%, and ≥90% within 2 days), the overall pattern of results was similar to the primary 70% or more treatment strategy. Intensive responses were associated with lower 14-day hospitalization risk at thresholds of 60% or more (RR, 0.78; 95% CI, 0.64–0.95; RD, −1.00%; 95% CI, −1.77 to −0.19) and 80% or more (RR, 0.74; 95% CI, 0.59–0.91; RD, −1.18%; 95% CI, −1.98% to −0.39%), whereas estimates at 50% or more and 90% or more were less precise and compatible with no difference. For 30-day hospitalization, estimates were directionally similar at some thresholds but generally more uncertain. Across all alternative thresholds, estimates for death remained close to null.

**Table 4.** Sensitivity Analyses of 14-Day and 30-Day Risks of Death and Hospitalization When Using Alternative Thresholds to Define the Intensive vs Nonintensive Antiviral Chemoprophylaxis Responses (Within 2 Days)

Threshold strategy and outcome	Follow-up	% (95% CI)			Risk ratio (95% CI)
		Nonintensive risk	Intensive risk	Risk difference	
≥50% vs <50%					
Death	14 d	1.68 (1.44 to 2.01)	1.53 (0.96 to 2.10)	−0.15 (−0.84 to 0.53)	0.91 (0.54 to 1.33)
	30 d	3.07 (2.61 to 3.63)	3.48 (2.15 to 4.75)	0.42 (−1.02 to 1.79)	1.14 (0.69 to 1.61)
Hospitalization	14 d	4.59 (4.11 to 5.18)	3.89 (3.13 to 4.58)	−0.70 (−1.67 to 0.10)	0.85 (0.66 to 1.02)
	30 d	7.32 (6.57 to 8.14)	7.45 (5.46 to 8.73)	0.14 (−1.99 to 1.56)	1.02 (0.74 to 1.23)
≥60% vs <60%					
Death	14 d	1.66 (1.43 to 1.98)	1.48 (0.84 to 2.31)	−0.18 (−0.89 to 0.72)	0.89 (0.50 to 1.45)
	30 d	3.03 (2.59 to 3.58)	3.02 (1.89 to 4.55)	−0.01 (−1.24 to 1.59)	1.00 (0.62 to 1.54)
Hospitalization	14 d	4.50 (4.02 to 5.07)	3.50 (3.00 to 4.14)	−1.00 (−1.77 to −0.19)	0.78 (0.64 to 0.95)
	30 d	7.25 (6.54 to 8.08)	5.90 (4.66 to 7.11)	−1.35 (−2.86 to 0.18)	0.81 (0.62 to 1.03)
≥80% vs <80%					
Death	14 d	1.65 (1.43 to 1.96)	1.76 (0.93 to 2.79)	0.11 (−0.78 to 1.19)	1.07 (0.54 to 1.74)
	30 d	3.00 (2.61 to 3.49)	3.72 (2.05 to 5.74)	0.72 (−0.99 to 2.92)	1.24 (0.68 to 2.01)
Hospitalization	14 d	4.51 (4.06 to 5.07)	3.32 (2.72 to 3.96)	−1.18 (−1.98 to −0.39)	0.74 (0.59 to 0.91)
	30 d	7.26 (6.56 to 8.05)	5.67 (4.23 to 7.09)	−1.59 (−3.19 to −0.03)	0.78 (0.57 to 1.00)
≥90% vs <90%					
Death	14 d	1.64 (1.41 to 1.90)	1.76 (1.09 to 2.85)	0.13 (−0.56 to 1.28)	1.08 (0.65 to 1.85)
	30 d	2.98 (2.61 to 3.45)	3.30 (1.91 to 5.31)	0.32 (−1.05 to 2.41)	1.11 (0.65 to 1.85)
Hospitalization	14 d	4.42 (3.99 to 4.98)	4.06 (2.74 to 5.58)	−0.37 (−1.70 to 1.10)	0.92 (0.62 to 1.26)
	30 d	7.26 (6.59 to 8.01)	7.69 (4.34 to 10.97)	0.43 (−2.98 to 3.80)	1.06 (0.59 to 1.53)

In analyses restricted to residents aged 65 years or older (eTable 4 in Supplement 1), estimates were directionally consistent with the primary analysis for 14-day hospitalization but less precise, reflecting a smaller sample (28 444 vs 35 086 resident trials; RR, 0.83; 95% CI, 0.67 to 1.01; RD, −0.76%; 95% CI, −1.56 to 0.03), while estimates for death and 30-day outcomes were close to the null with CIs spanning 1.

## Discussion

In this large retrospective cohort study applying the sequential target trial emulation framework and the randomize-censor-weight method to a novel NH EHR dataset, we found that residents in NHs who implemented chemoprophylaxis with oseltamivir for 70% or more within 2 days of outbreak detection experienced a considerable decrease in 14-day risk of hospitalization. An intensive chemoprophylaxis response to influenza outbreaks in NHs for alternative thresholds of 60% or more and 80% or more of residents also provided evidence that supports a reduction in the 14-day risk of hospitalization among NH residents. Therefore, national guidelines should recommend rapid chemoprophylaxis deployment within the first 2 days of an outbreak of at least 60% or more of all residents but more ideally 70% or more or 80% or more.

A prior cluster RCT of oseltamivir chemoprophylaxis reported reduced death or hospitalization but involved only 9 outbreaks and lacked statistical precision.^24^ Additional RCTs either did not evaluate death or hospitalization or were underpowered.^25^ Although 1 study found a 92% relative reduction in influenza infection with 6 weeks of seasonal oseltamivir prophylaxis, this approach differed fundamentally from postexposure prophylaxis. Notably, neither seasonal prophylaxis nor 6-week duration is currently recommended.^26^ Divergent findings across observational studies likely stem from differences in approaches to case identification and surveillance, outbreak definitions, chemoprophylaxis timing and extent, effect modifier distributions, and limited sample sizes. An Australian study using surveillance data found lower attack, hospitalization, and case fatality rates without prophylaxis, but delays between outbreak notification and prophylaxis initiation may have biased estimates.^27^ A target trial emulation was needed because chemoprophylaxis coverage is achieved over time and is intertwined with evolving outbreak conditions, making naive observational comparisons vulnerable to biases that arise from misaligning the timing of key elements of the study design as well as time-varying confounding.

Although guidelines allow chemoprophylaxis for residents on unaffected units, its broader impact is not well understood. The IDSA cites a lack of evidence-based data on chemoprophylaxis beyond outbreak-affected units.^2^ One study of 3 NH outbreaks reported the fewest hospitalizations when all residents received chemoprophylaxis,^10^ while others did not specify the extent of chemoprophylaxis,^28,29,30^ or focused on outbreak-affected units.^24^ While our results do not directly assess the effect on unaffected units, they support that administering chemoprophylaxis to most residents within 2 days of outbreak detection may help reduce hospitalizations. Our findings align with a modeling study showing a 36% reduction in hospitalizations when chemoprophylaxis followed current national guidelines,^18^ a Canadian study showing that a 1-day delay in oseltamivir initiation was associated with a 6% increased hospitalization risk,^31^ and a Taiwanese study documenting lower attack rates when chemoprophylaxis initiation began within 2 days.^32^

Our sensitivity analyses indicate that initiating chemoprophylaxis within 7 days of outbreak detection is unlikely to be fast enough to improve outcomes among residents. With the emergence of COVID-19 and the potential for cocirculation of other respiratory viruses, infection control practices in NHs have undergone renewed emphasis.^33,34^ Future work should identify which combinations and timing of outbreak-control measures (eg, respiratory precautions/masking, grouping residents with infection together, and chemoprophylaxis) are most effective for reducing viral transmission and improving clinically important outcomes in NHs. Additionally, trials comparing baloxavir marboxil to oseltamivir chemoprophylaxis in NHs are currently ongoing and may change recommendations if they demonstrate baloxavir’s noninferiority.^35,36^

### Limitations

This study has several potential limitations. First, we defined intensive chemoprophylaxis response at the NH level and therefore do not address all relevant clinical management questions for outbreaks. For instance, our EHR data did not reliably differentiate unit-level or wing-level locations, so we could not distinguish whether chemoprophylaxis was targeted to outbreak-affected units vs implemented facility-wide. Future work should explore more complex treatment strategies that incorporate detailed information on resident locations (eg, floorplans and unit maps). Second, a causal interpretation of our estimates assumes that the IPCW accounted for all important baseline and postbaseline covariates. This is particularly true in the intensive-strategy group, which experienced a large decrease in sample size during follow-up reflecting protocol-defined artificial censoring because many outbreaks did not achieve 70% or more coverage within the grace period. Certain factors, such as staff judgment and additional infection control practices, are difficult to measure, but we adjusted for several NH-level characteristics previously identified as associated with chemoprophylaxis utilization.^37^ We recognize that facilities that implement rapid and widespread chemoprophylaxis may differ in other infection control practices that could affect outcomes, such as use of masks, limiting admissions, and isolating affected residents. A large cluster-randomized trial would be preferred for identifying the causal effect of intensive chemoprophylaxis and standardizing other infection control practices, but such a trial is difficult to conduct during naturally occurring influenza outbreaks. Third, the randomize-censor-weight approach we implemented has been shown to provide valid point estimates; however, we could have implemented a clone-censor-weight approach, which reduces random variation and yields narrower CIs.^22^ Fourth, our results may be subject to other biases. While we accounted for censoring due to NHs deviating from their assigned treatment strategy, we did not estimate IPCW for censoring due to discharge to nonacute care settings, which was rare.^38^ We did not account for the competing risk of death when hospitalization was the outcome. We assumed that all missingness was at random, a strong assumption in practice, though we observed that measured baseline covariates were similarly distributed for resident-trials with any vs no missingness. The final years of our study overlapped with the COVID-19 pandemic, during which enhanced infection control practices may have influenced the effect size of chemoprophylaxis we observed. Lastly, although the eligibility criteria we specified in the target trial could introduce type 2 selection bias,^39^ the low percentage of outbreaks and residents excluded suggests any effect on our conclusion is minimal.

## Conclusions

In this study, we used a sequential target trial and randomize-censor-weight approach to emulate a hypothetical cluster-randomized pragmatic trial of influenza antiviral chemoprophylaxis in NHs. Our findings suggest intensive chemoprophylaxis of 70% or more of residents in response to influenza outbreaks in NHs within the first 2 days is associated with a lower 14-day risk of hospitalization among residents, with additional evidence to support a benefit when 60% or more or 80% or more of residents receive chemoprophylaxis. These findings could strengthen evidence-based recommendations to inform best practices in managing influenza outbreaks in NHs. Clinicians, administrators, and staff should consider rapid widespread chemoprophylaxis, including for residents in nonaffected units, as a strategy for containing future influenza outbreaks.

## Notes

Supplement 1. **eTable 1.** Abbreviated protocol of the hypothetical pragmatic cluster-randomized target trial and its emulation using observational data **eFigure 1.** Selection of eligible influenza outbreaks and eligible nursing home residents, 2018-19 to 2021-22 influenza seasons **eFigure 2.** Causal directed acyclic graph of the effect of an intensive nursing home antiviral chemoprophylaxis response on resident outcomes **eTable 2.** Distribution of nonstabilized inverse probability of censoring weights **eTable 3.** Observed (Unweighted) Cumulative Frequencies of Death and Hospitalization Outcome Events at 14 and 30 Days of Follow-up by Chemoprophylaxis Treatment Strategy **eTable 4.** Per-Protocol Analysis of 14-Day and 30-Day Risks of Death and Hospitalization Comparing Intensive (≥70% Within 2 Days) vs Non-Intensive Antiviral Chemoprophylaxis Responses Among Residents Aged 65 years and Older

Supplement 2.
Data Sharing Statement

## References

[1] Centers for Disease Control and Prevention. Interim guidance for influenza outbreak management in long-term care and post-acute care facilities. Influenza (Flu). September 26, 2024. Accessed April 23, 2025. https://archive.cdc.gov/#/details?url=https://www.cdc.gov/flu/hcp/infection-control/ltc-facility-guidance.html

[2] Uyeki TM, Bernstein HH, Bradley JS, . Clinical practice guidelines by the Infectious Diseases Society of America: 2018 update on diagnosis, treatment, chemoprophylaxis, and institutional outbreak management of seasonal influenza. Clin Infect Dis. 2019;68(6):e1-e47. doi:10.1093/cid/ciy866 30566567 PMC6653685

[3] Hayden FG, Belshe R, Villanueva C, . Management of influenza in households: a prospective, randomized comparison of oseltamivir treatment with or without postexposure prophylaxis. J Infect Dis. 2004;189(3):440-449. doi:10.1086/381128 14745701

[4] Johnston SL, Ferrero F, Garcia ML, Dutkowski R. Oral oseltamivir improves pulmonary function and reduces exacerbation frequency for influenza-infected children with asthma. Pediatr Infect Dis J. 2005;24(3):225-232. doi:10.1097/01.inf.0000154322.38267.ce 15750458

[5] Treanor JJ, Hayden FG, Vrooman PS, ; US Oral Neuraminidase Study Group. Efficacy and safety of the oral neuraminidase inhibitor oseltamivir in treating acute influenza: a randomized controlled trial. JAMA. 2000;283(8):1016-1024. doi:10.1001/jama.283.8.1016 10697061

[6] Whitley RJ, Hayden FG, Reisinger KS, . Oral oseltamivir treatment of influenza in children. Pediatr Infect Dis J. 2001;20(2):127-133. doi:10.1097/00006454-200102000-00002 11224828

[7] Nicholson KG, Aoki FY, Osterhaus AD, ; Neuraminidase Inhibitor Flu Treatment Investigator Group. Efficacy and safety of oseltamivir in treatment of acute influenza: a randomised controlled trial. Lancet. 2000;355(9218):1845-1850. doi:10.1016/S0140-6736(00)02288-1 10866439

[8] Welliver R, Monto AS, Carewicz O, ; Oseltamivir Post Exposure Prophylaxis Investigator Group. Effectiveness of oseltamivir in preventing influenza in household contacts: a randomized controlled trial. JAMA. 2001;285(6):748-754. doi:10.1001/jama.285.6.748 11176912

[9] Jester DJ, Peterson LJ, Dosa DM, Hyer K. Infection control citations in nursing homes: compliance and geographic variability. J Am Med Dir Assoc. 2021;22(6):1317-1321.e2. doi:10.1016/j.jamda.2020.11.010 33309701 PMC7834329

[10] Gorišek Miksić N, Uršič T, Simonović Z, . Oseltamivir prophylaxis in controlling influenza outbreak in nursing homes: a comparison between three different approaches. Infection. 2015;43(1):73-81. doi:10.1007/s15010-014-0703-4 25403263

[11] Bowles SK, Lee W, Simor AE, ; Oseltamivir Compassionate Use Program Group. Use of oseltamivir during influenza outbreaks in Ontario nursing homes, 1999-2000. J Am Geriatr Soc. 2002;50(4):608-616. doi:10.1046/j.1532-5415.2002.50153.x 11982659

[12] Parker R, Loewen N, Skowronski D. Experience with oseltamivir in the control of a nursing home influenza B outbreak. Can Commun Dis Rep. 2001;27(5):37-40. 11260987

[13] Bush KA, McAnulty J, McPhie K, ; Southern New South Wales Public Health Unit. Antiviral prophylaxis in the management of an influenza outbreak in an aged care facility. Commun Dis Intell Q Rep. 2004;28(3):396-400. doi:10.33321/cdi.2004.28.45 15574064

[14] Chang YM, Li WC, Huang CT, . Use of oseltamivir during an outbreak of influenza A in a long-term care facility in Taiwan. J Hosp Infect. 2008;68(1):83-87. doi:10.1016/j.jhin.2007.08.022 17945389

[15] Shijubo N, Yamada G, Takahashi M, Tokunoh T, Suzuki T, Abe S. Experience with oseltamivir in the control of nursing home influenza A outbreak. Intern Med. 2002;41(5):366-370. doi:10.2169/internalmedicine.41.366 12058885

[16] Centers for Disease Control and Prevention. Flu Season. Influenza (Flu). October 30, 2024. Accessed May 5, 2025. https://www.cdc.gov/flu/about/season.html

[17] Bhaskaran K, Smeeth L. What is the difference between missing completely at random and missing at random? Int J Epidemiol. 2014;43(4):1336-1339. doi:10.1093/ije/dyu080 24706730 PMC4121561

[18] Morris SE, Zipfel CM, Peer K, . Modeling the Clin Infect Dis. 2024;78(5):1336-1344. doi:10.1093/cid/ciad764 38072652 PMC11260992

[19] Cori A, Valleron AJ, Carrat F, Scalia Tomba G, Thomas G, Boëlle PY. Estimating influenza latency and infectious period durations using viral excretion data. Epidemics. 2012;4(3):132-138. doi:10.1016/j.epidem.2012.06.001 22939310

[20] Lansbury LE, Brown CS, Nguyen-Van-Tam JS. Influenza in long-term care facilities. Influenza Other Respir Viruses. 2017;11(5):356-366. doi:10.1111/irv.12464 28691237 PMC5596516

[21] Muller CJ, MacLehose RF. Estimating predicted probabilities from logistic regression: different methods correspond to different target populations. Int J Epidemiol. 2014;43(3):962-970. doi:10.1093/ije/dyu029 24603316 PMC4052139

[22] García-Albéniz X, Hernán MA, Logan RW, Price M, Armstrong K, Hsu J. Continuation of annual screening mammography and breast cancer mortality in women older than 70 years. Ann Intern Med. 2020;172(6):381-389. doi:10.7326/M18-1199 32092767

[23] García-Albéniz X, Hsu J, Hernán MA. The value of explicitly emulating a target trial when using real world evidence: an application to colorectal cancer screening. Eur J Epidemiol. 2017;32(6):495-500. doi:10.1007/s10654-017-0287-2 28748498 PMC5759953

[24] Booy R, Lindley RI, Dwyer DE, . Treating and preventing influenza in aged care facilities: a cluster randomised controlled trial. PLoS One. 2012;7(10):e46509. doi:10.1371/journal.pone.0046509 23082123 PMC3474842

[25] van der Sande MA, Meijer A, Şen-Kerpiclik F, . Effectiveness of post-exposition prophylaxis with oseltamivir in nursing homes: a randomised controlled trial over four seasons. Emerg Themes Epidemiol. 2014;11(1):13. doi:10.1186/1742-7622-11-13 25210532 PMC4159638

[26] Peters PH Jr, Gravenstein S, Norwood P, . Long-term use of oseltamivir for the prophylaxis of influenza in a vaccinated frail older population. J Am Geriatr Soc. 2001;49(8):1025-1031. doi:10.1046/j.1532-5415.2001.49204.x 11555062

[27] Merritt T, Hope K, Butler M, . Effect of antiviral prophylaxis on influenza outbreaks in aged care facilities in three local health districts in New South Wales, Australia, 2014. Western Pac Surveill Response J. 2016;7(1):14-20. doi:10.5365/wpsar.2015.6.3.005 27757249 PMC5052892

[28] Mahmud SM, Thompson LH, Nowicki DL, Plourde PJ. Outbreaks of influenza-like illness in long-term care facilities in Winnipeg, Canada. Influenza Other Respir Viruses. 2013;7(6):1055-1061. doi:10.1111/irv.12052 23145997 PMC4634272

[29] Monto AS, Rotthoff J, Teich E, . Detection and control of influenza outbreaks in well-vaccinated nursing home populations. Clin Infect Dis. 2004;39(4):459-464. doi:10.1086/422646 15356805

[30] Silva JB, Bosco E, Quilliam DN, Gravenstein S, Zullo AR. Antiviral chemoprophylaxis use during influenza outbreaks in Rhode Island long-term care facilities. J Am Med Dir Assoc. 2020;21(9):1354-1356. doi:10.1016/j.jamda.2020.05.020 32660853 PMC9015038

[31] Ye M, Jacobs A, Khan MN, . Evaluation of the use of oseltamivir prophylaxis in the control of influenza outbreaks in long-term care facilities in Alberta, Canada: a retrospective provincial database analysis. BMJ Open. 2016;6(7):e011686. doi:10.1136/bmjopen-2016-011686 27381211 PMC4947728

[32] Cheng HY, Chen WC, Chou YJ, Huang ASE, Huang WT. Containing influenza outbreaks with antiviral use in long-term care facilities in Taiwan, 2008-2014. Influenza Other Respir Viruses. 2018;12(2):287-292. doi:10.1111/irv.12536 29341490 PMC5820419

[33] Childs A, Zullo AR, Joyce NR, . The burden of respiratory infections among older adults in long-term care: a systematic review. BMC Geriatr. 2019;19(1):210. doi:10.1186/s12877-019-1236-6 31382895 PMC6683564

[34] Meehan A, Uth R, Gadbois EA, . Impact of COVID-19 on influenza and infection control practices in nursing homes. J Am Geriatr Soc. 2023;71(2):661-665. doi:10.1111/jgs.18061 36146903 PMC9538598

[35] Hayden FG, Sugaya N, Hirotsu N, ; Baloxavir Marboxil Investigators Group. Baloxavir marboxil for uncomplicated influenza in adults and adolescents. N Engl J Med. 2018;379(10):913-923. doi:10.1056/NEJMoa1716197 30184455

[36] Baloxavir versus oseltamivir for nursing home influenza outbreaks. Clinical Research Trial Listing. Accessed April 23, 2025. https://clinicaltrials.gov/study/NCT05012189?id=NCT05012189&rank=1

[37] Silva JBB. Examining infection control of respiratory viruses in nursing homes: COVID-19 and influenza. Brown University; 2024. Accessed April 30, 2025. https://repository.library.brown.edu/studio/item/bdr:b6fbv4d2/

[38] Cole SR, Hudgens MG, Brookhart MA, Westreich D. Risk. Am J Epidemiol. 2015;181(4):246-250. doi:10.1093/aje/kwv001 25660080 PMC4325680

[39] Lu H, Cole SR, Howe CJ, Westreich D. Toward a clearer definition of selection bias when estimating causal effects. Epidemiology. 2022;33(5):699-706. doi:10.1097/EDE.0000000000001516 35700187 PMC9378569

