# Supplementary material for: Prompt and Intensive Antiviral Chemoprophylaxis in Nursing Home Influenza Outbreaks
Source: JAMA Intern Med. 2026 Mar 30;186(6):714–22. doi: 10.1001/jamainternmed.2026.0401 (PMC13036633; doi:10.1001/jamainternmed.2026.0401)
Supplement: Supplement 1. — eTable 1. Abbreviated protocol of the hypothetical pragmatic cluster-randomized target trial and its emulation using observational data eFigure 1. Selection of eligible influenza outbreaks and eligible nursing home residents, 2018-19 to 2021-22 influenza seasons eFigure 2. Causal directed acyclic graph of the effect of an intensive nursing home antiviral chemoprophylaxis response on resident outcomes eTable 2. Distribution of nonstabilized inverse probability of censoring weights eTable 3. Observed (Unweighted) Cumulative Frequencies of Death and Hospitalization Outcome Events at 14 and 30 Days of Follow-up by Chemoprophylaxis Treatment Strategy eTable 4. Per-Protocol Analysis of 14-Day and 30-Day Risks of Death and Hospitalization Comparing Intensive (≥70% Within 2 Days) vs Non-Intensive Antiviral Chemoprophylaxis Responses Among Residents Aged 65 years and Older [file jamainternmed-e260401-s001.pdf]

Supplemental Online Content

Silva JB, Hsieh HT, Howe CJ, Gravenstein S, Reich LA, Zullo AR. Prompt and intensive antiviral chemoprophylaxis in nursing home influenza outbreaks. *JAMA Intern Med.* Published online March 30, 2026.
doi:10.1001/jamainternmed.2026.0401

eTable 1. Abbreviated protocol of the hypothetical pragmatic cluster-randomized target trial and its emulation using observational data

eFigure 1. Selection of eligible influenza outbreaks and eligible nursing home residents, 2018-19 to 2021-22 influenza seasons

eFigure 2. Causal directed acyclic graph of the effect of an intensive nursing home antiviral chemoprophylaxis response on resident outcomes

eTable 2. Distribution of nonstabilized inverse probability of censoring weights

eTable 3. Observed (Unweighted) Cumulative Frequencies of Death and Hospitalization Outcome Events at 14 and 30 Days of Follow-up by Chemoprophylaxis Treatment Strategy

eTable 4. Per-Protocol Analysis of 14-Day and 30-Day Risks of Death and Hospitalization Comparing Intensive (≥70% Within 2 Days) vs Nonintensive Antiviral Chemoprophylaxis Responses Among Residents Aged 65 years and Older

This supplemental material has been provided by the authors to give readers additional information about their work.

© 2026 Silva JB et al. *JAMA Internal Medicine*.

eTable 1. Abbreviated protocol[1-3] of the hypothetical pragmatic cluster-randomized target trial and its emulation using observational data.

Protocol component	Hypothetical target pragmatic cluster-randomized trial	Observational study emulating the hypothetical target trial
**1. Eligibility**	*Nursing home eligibility*:<br>• Influenza outbreak detected (baseline)<br>o Date of incident influenza cases identified within 72 hours<br>• ≥30 residents in nursing home at baseline<br>• No missing facility characteristics, and no missing MDS covariate information for >50% of residents<br><br>*Resident eligibility*:<br>• Residence in eligible nursing home for at least one day<br>• Age ≥18 years<br>• Complete covariate information	• Same as the target trial, but nursing homes required to have an actively reporting electronic medication administration record at baseline and no influenza outbreak in the two weeks prior to baseline<br>• Residents aged 18-110
**2. Treatment strategies**	• *Intensive antiviral chemoprophylaxis response:*<br>Initiation of antiviral chemoprophylaxis with oseltamivir for ≥70% of eligible residents within 2 days of baseline.<br>• *Nonintensive antiviral chemoprophylaxis response:* Do not initiate antiviral chemoprophylaxis with oseltamivir for ≥70% of eligible residents and abstain from doing so during 30 days of follow-up.<br>• Under both treatment strategies, the decision to implement any additional infection control interventions at the nursing home level is left to the discretion of nursing home clinicians, staff, and administrators. At the resident level, the decision to initiate other therapies besides oseltamivir is left at the discretion of residents, their caregivers, and their clinicians.	• Same as target trial, but treatment was ascertained from medication administration records.<br>• Nursing homes and residents were classified according to the strategy that their data were compatible with.
**3. Treatment Assignment**	• Facilities are randomized to a treatment strategy at baseline without blinding. Nursing home staff and administrators are potentially aware of the assigned treatment strategy. The lack of blinding (i.e., open-label nature) of the trial is due to the reality	• Nursing homes are assumed randomized to their treatment strategy conditional on

© 2026 Silva JB et al. *JAMA Internal Medicine*.

	that nursing home facility-wide implementation of chemoprophylaxis cannot realistically be masked from nursing home clinicians, staff, or administrators.<br>• Eligible residents are assigned the treatment strategy of the nursing home they reside in and are unlikely to be aware of the treatment strategy at the nursing home level.	the following baseline variables: tertile of Alzheimer's disease and related dementias prevalence, tertile of the count of prior influenza tests ordered during the week prior to baseline, whether the outbreak was first to occur in the nursing home during a given season, and whether additional cases of influenza had been identified.
**4. Follow-up**	• Residents are followed from baseline (point of randomization) until the first occurrence of an outcome event (each evaluated separately), discharge from the nursing home to a location other than an acute care hospital, or the end of follow-up 30 days after baseline.	• Same as target trial.
**5. Outcomes**	• All-cause mortality<br>• All-cause hospitalization.	• Same as target trial, except deaths occurring outside the nursing home are not reported.
**6. Causal contrasts of interest**	• Intention-to-treat effect<br>• Per-protocol effect	• Observational analogue of the per-protocol effect.
**7. Analysis**	• Per-protocol effect: Estimate:<br>o 14-day and 30-day risk, risk differences, risk ratios, and survival curves comparing the treatment	• Same as target trial, except nursing homes are eligible to

© 2026 Silva JB et al. *JAMA Internal Medicine*.

	groups. Use nonparametric cluster bootstrapping at the nursing home level with 500 resamplings to calculate percentile-based 95% confidence intervals for all estimates.<br>o Residents are censored when their data stop being compatible with their assigned treatment strategy. Each resident receives an inverse probability of censoring weight that is estimated from the data to adjust for potential selection bias introduced by censoring.	participate in multiple trials.

The summary of the target trial protocol was specified in accordance with the following three publications offering guidance on how to specify the target trial and its emulation: 1. Cashin AG, Hansford HJ, Hernan MA, et al. Transparent Reporting of Observational Studies Emulating a Target Trial-The TARGET Statement. *JAMA*. Sep 23 2025;334(12):1084-1093. doi:10.1001/jama.2025.13350; 2. Hernan MA, Robins JM. Using Big Data to Emulate a Target Trial When a Randomized Trial Is Not Available. *Am J Epidemiol*. Apr 15 2016;183(8):758-64. doi:10.1093/aje/kwv254; and 3. Hernan MA, Wang W, Leaf DE. Target Trial Emulation: A Framework for Causal Inference From Observational Data. *JAMA*. Dec 27 2022;328(24):2446-2447. doi:10.1001/jama.2022.21383.

© 2026 Silva JB et al. *JAMA Internal Medicine*.

eFigure 1. Selection of eligible influenza outbreaks and eligible nursing home residents, 2018-19 to 2021-22 influenza seasons.

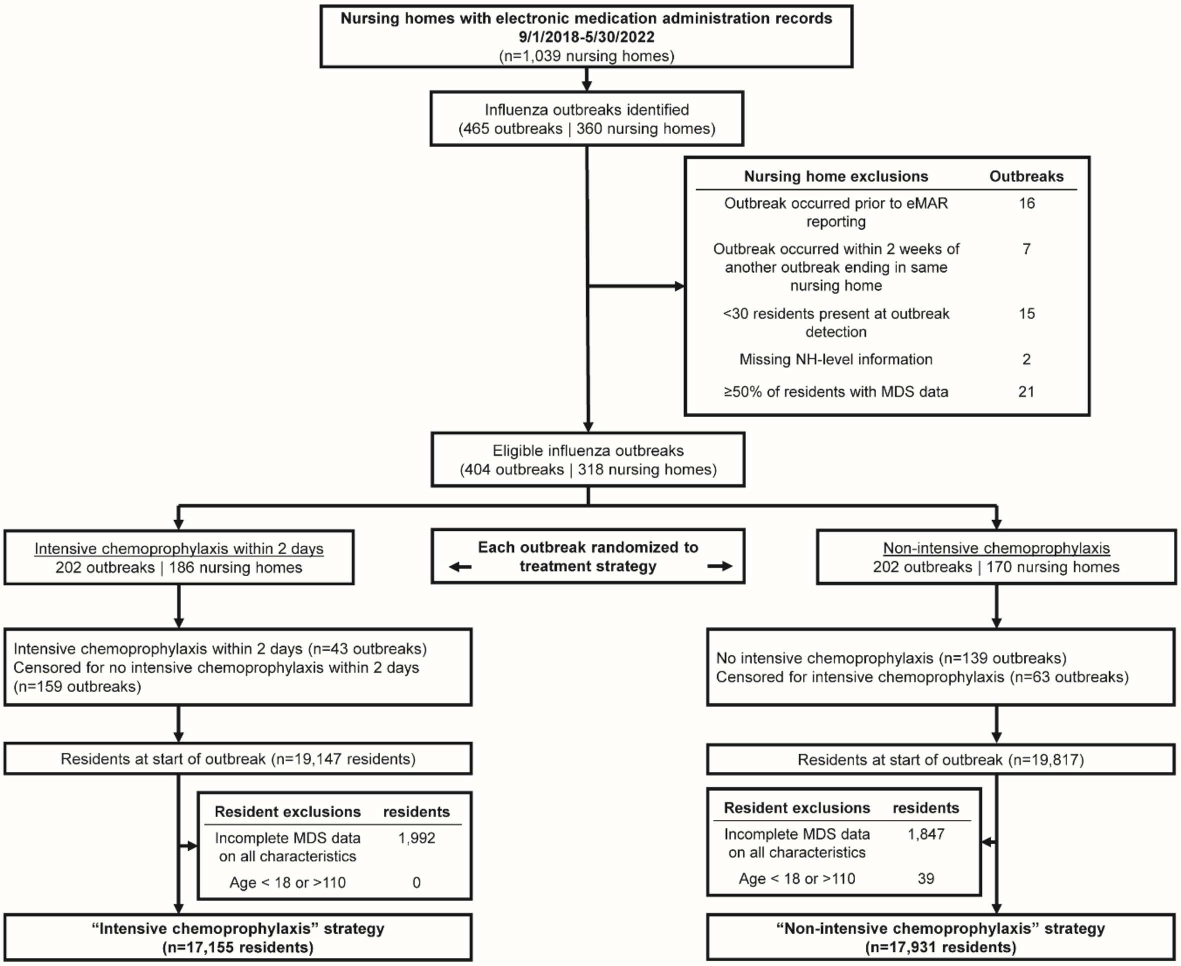

Abbreviations: eMAR, electronic medication administration record; NH, nursing home; MDS, Minimum Data Set.

© 2026 Silva JB et al. *JAMA Internal Medicine*.

eFigure 2. Causal directed acyclic graph of the effect of an intensive nursing home antiviral chemoprophylaxis response on resident outcomes.

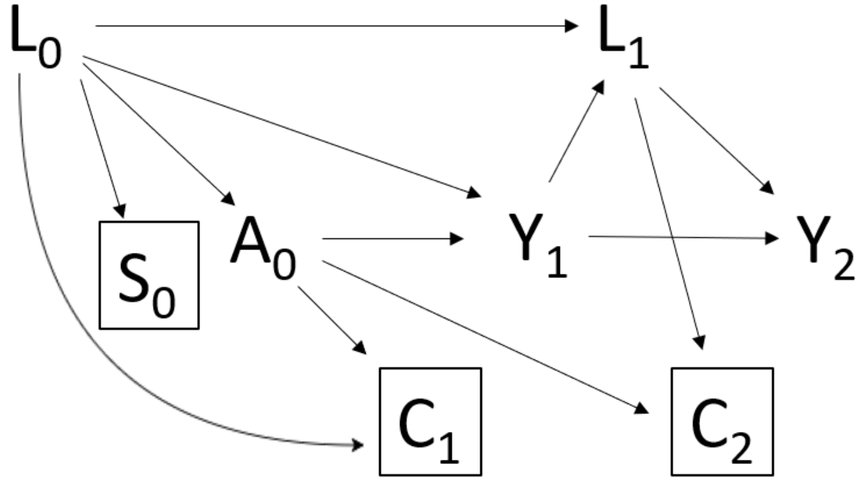

Where:

A= assignment to treatment strategy (i.e., intensive versus nonintensive antiviral chemoprophylaxis response)
S = selection into the analytic sample (i.e., meeting eligibility criteria at resident and nursing home level)
C = censoring due to deviation from the treatment strategy
L = vector of nursing home level covariates
Y = outcome (i.e., death, hospitalization)

Legend:

The directed acyclic graph (DAG) depicts the assumed causal structure used to estimate the per-protocol effect of an intensive versus nonintensive antiviral chemoprophylaxis treatment strategy using the randomize-censor-weight approach. L_0 denotes baseline covariates measured at outbreak detection (time zero). S_0 denotes selection into the analytic cohort (by meeting eligibility and having complete baseline data). A_0 denotes the nursing home-level treatment strategy assigned at baseline (intensive versus nonintensive antiviral chemoprophylaxis). Y_1 and Y_2 denote the outcomes evaluated over successive follow-up intervals (i.e., through 14 and 30 days). L_1 denotes time-varying covariates measured during follow-up that may be affected by prior treatment history and may influence subsequent outcomes and censoring. C_1 and C_2 denote censoring indicators for deviation from the assigned strategy during each interval. Arrows indicate assumed direct causal relationships, and inverse probability of censoring weighting targets the per-protocol estimand by accounting for measured predictors of censoring and outcomes.

© 2026 Silva JB et al. *JAMA Internal Medicine*.

eTable 2. Distribution of nonstabilized inverse probability of censoring weights.

Outcome	Strategy	Mean	Standard deviation	Minimum	Maximum
Death	Nonintensive	1.3	0.4	1.0	4.8
	Intensive	4.3	5.4	1.0	20.2
Hospitalization	Nonintensive	1.3	0.4	1.0	4.7
	Intensive	4.3	5.4	1.0	20.2

Pooled logistic regression models adjusted for baseline tertile of ADRD prevalence, tertiles of the count of prior influenza tests ordered during the week prior to the outbreak, whether the outbreak was first to occur in the NH during a given season, and a time-varying indicator of whether additional cases of influenza had been identified, beyond the initial 2 cases. To allow for predicted probabilities to change over time, the model for the nonintensive group also included time and interactions between time and all other characteristics. The weight distribution is for the primary analysis comparing administering chemoprophylaxis with oseltamivir to ≥70% of eligible residents within 2 days of outbreak detection versus providing no chemoprophylaxis or chemoprophylaxis for more than 0% but less than 70% of residents.

© 2026 Silva JB et al. *JAMA Internal Medicine*.

eTable 3. Observed (Unweighted) Cumulative Frequencies of Death and Hospitalization Outcome Events at 14 and 30 Days of Follow-up by Chemoprophylaxis Treatment Strategy.

Outcome	Follow-Up	Nonintensive Strategy, Number of Events	Intensive Strategy, Number of Events
Death	14 days	210	70
	30 days	354	108
Hospitalization	14 days	606	186
	30 days	907	272

© 2026 Silva JB et al. *JAMA Internal Medicine*.

eTable 4. Per-Protocol Analysis of 14-Day and 30-Day Risks of Death and Hospitalization Comparing Intensive (≥70% Within 2 Days) vs Nonintensive Antiviral Chemoprophylaxis Responses Among Residents Aged 65 years and Older.

Outcome	Follow-Up	Nonintensive risk % (95% CI)	Intensive risk % (95% CI)	Risk difference (%) (95% CI)	Risk ratio (95% CI)
Death	14 days	1.88 (1.59, 2.23)	1.67 (1.12, 2.85)	-0.20 (-0.94, 0.95)	0.89 (0.56, 1.55)
	30 days	3.43 (2.97, 3.99)	3.64 (2.46, 5.76)	0.20 (-1.14, 2.30)	1.06 (0.68, 1.68)
Hospitalization	14 days	4.36 (3.87, 4.95)	3.60 (2.98, 4.20)	-0.76 (-1.56, 0.03)	0.83 (0.67, 1.01)
	30 days	7.05 (6.39, 7.92)	6.29 (4.68, 7.40)	-0.76 (-2.57, 0.39)	0.89 (0.64, 1.06)

Abbreviations: CI, confidence interval.

© 2026 Silva JB et al. *JAMA Internal Medicine*.
