# Supplementary material for: Prompt and Intensive Antiviral Chemoprophylaxis in Nursing Home Influenza Outbreaks
Source: JAMA Intern Med. 2026 Mar 30;186(6):714–22. doi: 10.1001/jamainternmed.2026.0401 (PMC13036633; doi:10.1001/jamainternmed.2026.0401)
Supplement: Supplement 2. — Data Sharing Statement [file jamainternmed-e260401-s002.pdf]

Data Sharing Statement

Silva. Prompt and Intensive Antiviral Chemoprophylaxis in Nursing Home Influenza Outbreaks. *JAMA Intern Med*. Published March 30, 2026. doi:10.1001/jamainternmed.2026.0401

Data
Data available: Yes
Data types: Other (please specify)
Additional Information: The findings are based on data obtained through a partnership between the Brown University School of Public Health and 12 U.S. long-term care facility chains. The proprietary data were provided to Brown University for the purpose of conducting research on COVID-19 and respiratory viruses more broadly. These data were the precursor to what would become the National Institute on Aging-funded Long-Term Care Data Cooperative (LTCDC). While the data employed in the current study are not the same as the LTCDC data and are not publicly available, the LTCDC data are similar in structure and are now publicly available. Researchers interested in using the LTCDC data can apply to use it at the following website: https://www.ltcdatacooperative.org.
How to access data: The findings are based on data obtained through a partnership between the Brown University School of Public Health and 12 U.S. long-term care facility chains. The proprietary data were provided to Brown University for the purpose of conducting research on COVID-19 and respiratory viruses more broadly. These data were the precursor to what would become the National Institute on Aging-funded Long-Term Care Data Cooperative (LTCDC). While the data employed in the current study are not the same as the LTCDC data and are not publicly available, the LTCDC data are similar in structure and are now publicly available. Researchers interested in using the LTCDC data can apply to use it at the following website: https://www.ltcdatacooperative.org.
When available: With publication

Supporting Documents
Document types: None

Additional Information
Who can access the data: Any researchers interested in using the data can apply to use it at the following website: https://www.ltcdatacooperative.org.
Types of analyses: The types of analyses for which the data can be made available is governed by the Long-Term Care Data Cooperative. Researchers can submit all types of analyses for approval at https://www.ltcdatacooperative.org.
Mechanisms of data availability: Researchers interested in using the data must following the most recent mechanisms established by the Long-Term Care Data Cooperative and documented on the following website: https://www.ltcdatacooperative.org.
